# A Rare Case of Fatal Thyroid Hemorrhage After Fine-Needle Aspiration: Case Report and Review of the Literature

**DOI:** 10.1097/PAF.0000000000000761

**Published:** 2022-08-04

**Authors:** Alessandro Bonsignore, Martina Drommi, Francesca Frigiolini, Anna Roncallo, Francesco Ventura, Francesca Buffelli, Ezio Fulcheri

**Affiliations:** From the ∗ Department of Legal and Forensic Medicine, University of Genova; † IRCCS—Ospedale Policlinico San Martino; ‡ Clinical Pathology Unit, IRCCS—Istituto Giannina Gaslini; § Department of Pathology, San Martino Hospital, University of Genova, Genova, Italy.

**Keywords:** thyroid nodule, fine-needle aspiration, fatal hemorrhage, sudden death, forensic pathology, autopsy

## Abstract

Sudden death due to massive hemorrhage after a mini-invasive ambulatory diagnostic procedure is extremely rare. Fine-needle aspiration (FNA) of thyroid nodules is very safe, displaying a low rate of complications, all of which mild and often self-limiting. In few cases do these complications necessitate surgical decompression, and rarely does FNA of a thyroid nodule lead to the death of the patient.

We report a case of sudden death caused by respiratory insufficiency after compression of the vascular and nervous structures of the neck and obstruction of the upper airways by hemorrhages dissecting the thyroidal and perithyroidal tissues in a 78-year-old woman. These hemorrhages were the result of vascular lacerations caused during diagnostic FNA of a nodule suspected of malignancy. In such cases, it is important to conduct a complete autopsy and histological analysis to ascertain the origin of massive hemorrhage involving the structures of the neck and to attribute the cause of death to the aforementioned procedure. The forensic pathologist must bear in mind that even extremely small damage, such as that produced by a fine needle, may cause a fatal hemorrhage in subjects with a subverted anatomo-pathological picture (such as, for example, the massive fibrosis of an organ).

Fine-needle aspiration (FNA) biopsy of a thyroid nodule is a mini-invasive diagnostic procedure used to confirm or exclude the malignancy of the nodule. Nodular disease is characterized by the disordered growth of thyroid cells often associated to the gradual development of fibrosis, areas of hemorrhage, or lymphocyte infiltration; it affects about 3% to 7% of adults who undergo objective examination.^1^

Current guidelines on the diagnosis and management of thyroid nodules^2,3^ recommend that FNA biopsy be carried out on capsular or paratracheal nodular lesions, those suspected of lymph-node or extrathyroidal involvement, and those in patients with a positive personal or family history of thyroid carcinoma or with consistent clinical symptoms (dysphonia); moreover, the nodules must present marked hypoecogenicity, lobulated irregular margins, anomalous vascularization, and calcifications, in addition to having a diameter of more than 10 mm.

Complications of FNA procedures are extremely rare. Those described in the literature are the following: pain at the site of intervention, infections, swelling of the neck, and the formation of small hematomas inside the gland, which are spontaneously reabsorbed within a few days.^2^ Particular caution should be exercised, however, in patients on anticoagulant therapy.^3,4^ Indeed, in a study of 593 patients who underwent FNA, hematomas were documented in 2 of the 144 patients who were on anticoagulant therapy, whereas, in the 449 patients who were not taking anticoagulants, 4 episodes of bleeding were observed.^5^

An even rarer complication is that of massive hemorrhage associated to the rapid onset of dyspnea due to deviation and compression of the trachea and requiring emergency surgical decompression.^6–14^ Moreover, cases of death after FNA are practically unknown in the literature.^15,16^

Here, we present the case of a woman affected by multinodular goiter who underwent diagnostic FNA biopsy of a thyroid nodule. After the procedure, she suffered a massive hemorrhage of the thyroidal and perithyroidal tissues, which caused cyanosis and worsening dyspnea; she died 6 hours later.

## CASE REPORT

### Case History

A 78-year-old woman, who was obese (body mass index, 33.4 kg/m^2^) and affected by multinodular goiter, underwent medical examinations, including thyroid echography, which revealed an increase in the size of some nodules. The endocrinologist suggested that an FNA biopsy be taken of a nodule that presented also inhomogeneous ultrasound appearance and internal vascularization.

During the FNA procedure, which was performed under ultrasound guidance, no problems arose. About 30 minutes after the end of the procedure, the patient, who was not taking antiplatelet/anticoagulant drugs, left the outpatient clinic and went to lunch. During her meal, the woman began to feel discomfort in her neck. Once back home, she took an analgesic and lay down to rest. After about 2 hours, however, the woman began to suffer from swelling of the neck, dysphonia, and worsening dyspnea. The general practitioner was alerted, and the woman was promptly taken to the emergency department; although resuscitation maneuvers (cardiopulmonary resuscitation, tracheal intubation, central venous cannulation, and advanced medication administration) were carried out for about 40 minutes, the patient was pronounced dead 6 hours after the FNA procedure.

### Autopsy and Histological Findings

The public prosecutor ordered an autopsy to ascertain the cause of death and to investigate any hypothesis of professional liability on the part of the doctor who had performed the FNA. External examination revealed a large swelling, of soft consistency, in the anterior cervical region, leakage of blood from the mouth, and the marks of needle puncture on the right wrist and in the fold of the left elbow. The rest of the external examination was unremarkable.

**FIGURE 1:**
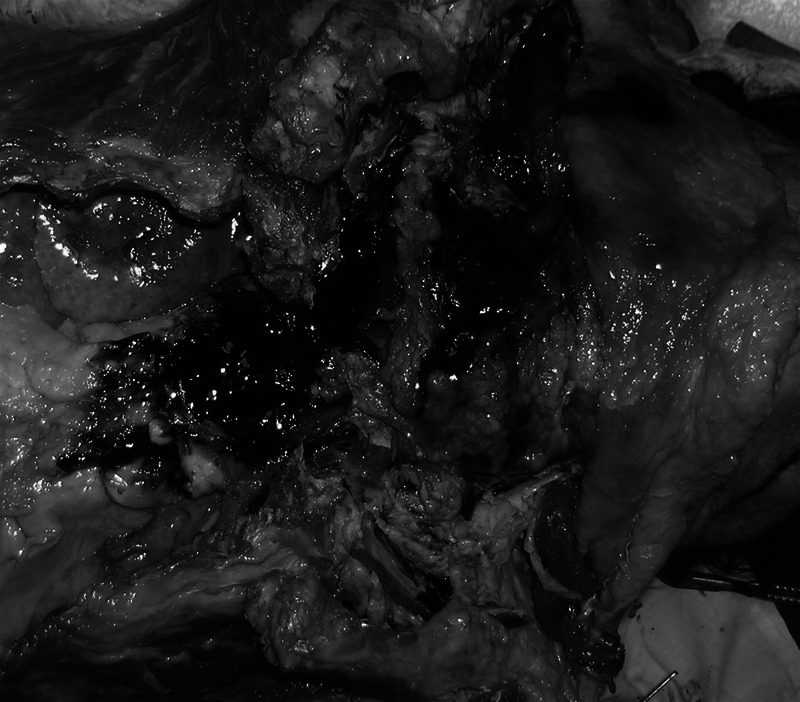
Massive hemorrhagic infarct involving the neck muscles and subcutaneous tissue of the supraclavicular region, especially on the right.

On section, a massive hematoma within the neck muscles and subcutaneous tissue of the supraclavicular region, especially on the right, was noted. At the right side of the suprasternal notch, at the base of the neck, an abundant collection of blood was found (Fig. 1).

The trachea presented blood leakage on compression, and coagulated blood was found along its entire length; the macroscopic architecture of the thyroid was completely disrupted by the hemorrhage. The lungs, which were congested and edematous, leaked pinkish foam when pressed, whereas the remaining visceral organs were markedly anemic but unremarkable. The heart weighted 400 g; the coronary arteries were elastic, and there were no signs of atherosclerosis.

**FIGURE 2:**
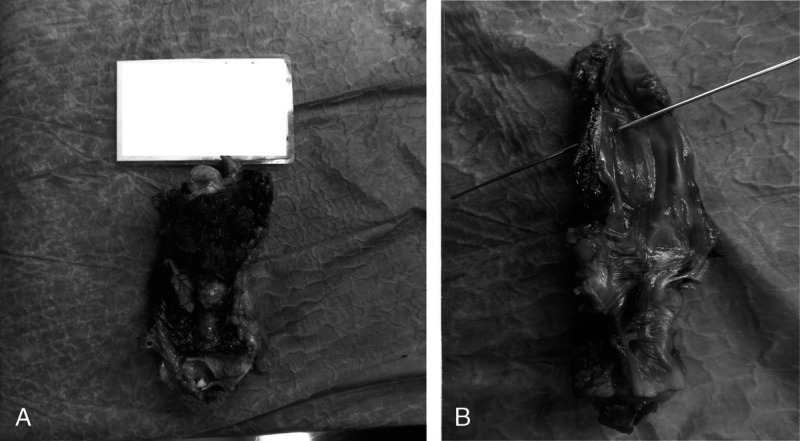
Organs of the neck after fixation in formalin. A, Area of hemorrhagic infiltration at the level of the supraglottic plane, surrounding the right lateral region of the esophagus. B, Interruption of a few millimeters along the vertical axis of the right jugular vein.

After fixation in formalin, sampling of the organs of the neck was carried out. During this operation, a 3 × 15 cm area of hemorrhagic infiltration was observed at the level of the supraglottic plane, in the right lateral region of the esophagus (Fig. 2A). Posterior to the course of the trachea, a large quantity of coagulated blood was seen, whereas the right jugular vein presented an interruption of a few millimeters along the vertical axis (Fig. 2B). Eight samples were taken, one of which included the vein at its presumed point of rupture, whereas the others consisted of sections of the neck region affected by the hemorrhagic infarct.

**FIGURE 3:**
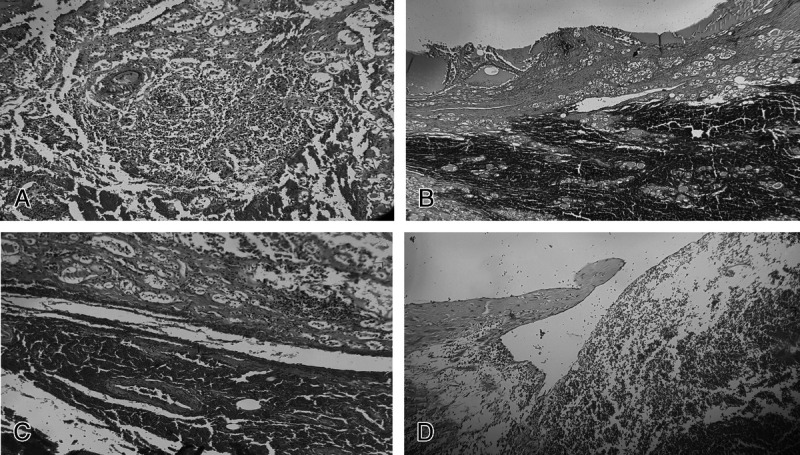
Histological findings. A, Granulomatous process with foreign body-type giant cells, reactive to colloid extravasation and microhemorrhages in the thyroid gland (hematoxylin and eosin [H&E], ×20). B, Marked hemorrhagic extravasations in the thyroid tissues (H&E, ×20). C, A medium-caliber vein of the perithyroid tissues with lacerated wall (H&E, ×20). D, Laceration of small thickness of the jugular vein, in the absence of inflammatory reaction (H&E, ×20).

Histological analysis revealed a thyroid architecture characterized by multiple colloidocystic nodes and a structure disrupted by a fibrosclerotic process constituted by hyaline connective tissue arranged in bundles that tended to segment the gland. In addition, a granulomatous process with foreign body-type giant cells, reactive to colloid extravasation and microhemorrhages, was seen (Fig. 3A). The overall picture was characterized by ubiquitous recent hemorrhages, with more marked hemorrhagic extravasations in the perithyroid tissues and in the lobules of adipose tissue (Fig. 3B); in this setting, 3 medium-caliber veins with lacerated walls, apposition of fibrin meshes, and reactive lymphogranulocytic infiltrate were found (Fig. 3C).

With regard to the full-thickness lesion of the jugular vein, histology revealed a laceration of small thickness (2 mm) in the absence of inflammatory reaction (granulocytic or lymphocytic elements in the vessel wall); moreover, the vein appeared dystrophic, with marked thinning and loss of the normal smooth muscle component of the wall (Fig. 3D).

The cause of death was attributed to the compression of the vascular and nervous structures of the neck and obstruction of the upper airways, secondary to abundant dissecting hemorrhages in the thyroidal and perithyroidal tissues due to vascular lesions of the adjacent veins after FNA of the thyroid nodule.

## DISCUSSION

Here, we report a rare case of death due to massive hemorrhage of the thyroid parenchyma and of the perithyroidal tissues after an FNA procedure.

Multinodular nontoxic goiter is a chronic disease characterized by enlargement of the thyroid in the absence of neoplastic alterations. The enlarged gland typically presents macroscopic irregular nodules. Histological examination of the nodules reveals flattened or cuboid epithelial lining cells and, occasionally, papillae that protrude into the follicular lumen. The frequent presence of hemosiderin deposits and cholesterol granulomas is indicative of a previous hemorrhage. Single follicles or clusters of follicles separated by dense fibrosis with dystrophic calcifications may also be observed. Hemorrhage and chronic inflammation are frequent.^17,18^

The development of a massive hemorrhage capable of determining compression of the vascular-nervous structures of the neck and obstruction of the upper airways, threatening the life of the patient, is an extremely rare complication of needle aspiration procedures of nodules in a context of a multinodular goiter. Indeed, although the thyroid is a richly vascularized organ, bleeding of the parenchyma and the surrounding tissues is a sporadic event and is generally self-limiting, causing only mild symptoms. Fatal cases are extremely rare.

A thorough review of the literature reveals that only 12 cases of thyroid hemorrhages after needle aspiration procedures have been described (Table 1). This complication proved fatal in only 2 cases.^15,16^

**TABLE 1:** Cases of Thyroid Hemorrhages After Needle Aspiration Procedures According to the Literature

	Authors	Sex; Age, y	Thyroid Disease	Symptoms Reported After FNA	Type of Complication	Anticoagulant or Antiplatelet Drugs	Death of the Patient
Case 1	Katagiri et al^6^	F, 47	Nodule	Neck swelling, pain, dyspnea	Massive hematoma	No	No
Case 2	Noordzij and Goto^7^	F, 60	Lymphocytic thyroiditis	Dyspnea, pain, ecchymosis, neck swelling	4 × 5 × 7 cm Hematoma	No	No
Case 3	Yoshida et al^8^	F, 62	Hypervascular nodule	Neck swelling, pain	Hemorrhage inside thyroid lobes	No	No
Case 4	Donatini et al^9,14^	F, 74	2-cm Isthmic nodule	Neck swelling, hoarseness, dyspnea	Hematoma, bleeding of ima artery	No	No
Case 5	Donatini and Masoni^9^	F, 68	2.5-cm Isthmic nodule	Neck swelling, dyspnea, hoarseness, dysphonia	Bleeding of the inferior thyroid vein	No	No
Case 6	Donatini and Masoni^9^	F, 72	15-mm Lymphnode	Neck swelling, dyspnea	Partial laceration of the left lobe	No	No
Case 7	Park and Yoon^10^	F, 72	Nodule	Dyspnea, neck swelling, ecchymosis	Anterior hematoma	No	No
Case 8	Lee et al^11^	F, 45	Nodule	Pain, neck swelling, mild dyspnea	Intrathyroid hemorrhage	No	No
Case 9	Roh et al^12^	F, 55	Bilateral multiple nodules, Hashimoto's thyroiditis	Pain, dysphagia, neck swelling	Parenchymal hemorrhage	No	No
Case 10	Hor and Lahiri^13^	F, 62	Nodules	Dysphonia, tachypnea	Intrathyroid hemorrhage	Aspirin	No
Case 11	Kakiuchi et al^15^	F, 68	Mass lesion	Found dead	Massive hemorrhage of the left lobe	No	Yes
Case 12	Strachan et al^16^	F, 79	Large nodule on the isthmus	Cough and dyspnea	Hemorrhage within the capsule of the thyroid	Aspirin	Yes

Specifically, Kakiuchi et al^15^ reported the case of a 68-year-old woman who underwent FNA to investigate the nature of a mass involving the left lobe of the thyroid. After the procedure, she returned home and was found dead about 6.5 hours later. Autopsy revealed a large laryngeal edema and a massive hemorrhage on the anterior and lateral surfaces of the gland. Death was attributed to obstruction of the upper airways due to the massive perithyroidal hemorrhage.

The second fatal case was reported by Strachan et al.^16^ This involved a 79-year-old woman who was affected by a voluminous thyroid nodule that extended to the upper mediastinum and which was in contact with the anterior wall of the trachea. After an FNA procedure, she died before reaching the emergency department. Autopsy indicated an acute hemorrhage originating inside the thyroid gland as the cause of death.

Both cases involved elderly women (older than 65 years): this population, because of an increased venous fragility, is at greater risk of developing fatal vascular complications during routine procedures.

In the remaining cases reported in the literature, bleeding was less severe, and prompt surgical decompression was able to save the patients' lives.

Two possible mechanisms responsible for bleeding have been described: anomalous vascularization of a multinodular goiter, which increases the fragility of the vessels and hence the probability of their rupture, and the creation of arteriovenous shunts in the setting of thyroid parenchyma with disrupted architecture.^19,20^

In the case reported here, disruption of the parenchymal structure was observed. Indeed, histological examinations revealed a colloid nodular goiter with severe fibrosclerotic sequelae secondary to previous thyroiditis. This condition had weakened the thyroid vessels, which appeared dystrophic. A minimal trauma, caused by the needle aspiration procedure, was therefore sufficient to trigger a progressive intrathyroidal bleeding.

Moreover, the histological findings corroborated this hypothesis. The hemorrhagic infarct, in the thyroidal and perithyroidal areas and in the vicinity of the adjacent lacerated veins, displayed margination and the interposition of reactive lymphogranulocytic elements, which typically appear 5 to 6 hours after a hemorrhagic trauma. This time lapse is therefore consistent with the circumstantial data, which attest that the FNA procedure was carried out at 11.00 am and that the patient died at 5.30 pm.

Thus, within a few hours, the hemorrhage swelled the volume of the gland, causing external compression of the vascular-nervous structures of the neck and acute obstruction of the upper airways, leading to respiratory insufficiency.

With regard to the jugular vein lesion observed during autopsy examination, histological examination would be more consistent with the injury to not have occurred synchronous with the rupture of the thyroid vessels, given the absence of intralesional and perilesional granulocytic inflammatory reaction (as was documented in the thyroid tissues). A jugular vein lesion occurred during FNA of thyroid nodule would determine an inflammatory reaction with apposition of fibrin meshes and reactive lymphogranulocytic infiltrate. Furthermore, the woman was taken to the emergency department where resuscitation maneuvers, including central venous cannulation, were carried out.

These elements indicate that the laceration of the jugular vein occurred during resuscitation maneuvers carried out in limine vitae.

The present article describes an extremely rare complication of a needle aspiration procedure; indeed, only 2 other fatal cases are reported in the literature.

In this particularly complex case, only careful and complete investigation of the circumstantial data and autopsy and histological findings enabled the correct diagnosis to be made. The fixation and histological examination of the specimen affected by the hemorrhage proved to be of fundamental importance. In addition to identifying the site of bleeding, they enabled a differential diagnosis to be made between lesions caused by needle aspiration and those caused by resuscitation maneuvers.

The case reported is not only of medicolegal interest but also of clinical relevance. First of all, operators who perform needle aspiration procedures should always inform their patients of the hemorrhagic risk, albeit minimal. Equally important are the knowledge and application of the guidelines, to prevent the occurrence of complications. These precautions are also important in terms of avoiding medicolegal disputes. Particular caution should be exercised when dealing with subjects who present risk factors. In such cases, it is advisable to carry out detailed preoperative tests, prolong the observation period after the procedure, and, at the moment of discharge, carefully instruct the patient as to the alarm symptoms of a possible ongoing hemorrhage. Prompt recognition of hemorrhagic complications increases the possibility of therapeutic intervention and, therefore, of survival.^6,9,12,13^

From the medicolegal standpoint, the case did not have penal repercussions, in that the public prosecutor deemed that the operator who performed the FNA was not liable to penal prosecution, on the grounds that there was no clear evidence of culpable conduct. In the civil court, however, the patient's family members were awarded damages, given that the causal link between the procedure and the patient's death was established within the terms of the preponderant scientific evidence.
